# Activity in the lateral occipital cortex between 200 and 300 ms distinguishes between physically identical seen and unseen stimuli

**DOI:** 10.3389/fnhum.2012.00211

**Published:** 2012-07-25

**Authors:** Ying Liu, Anne-Lise Paradis, Lydia Yahia-Cherif, Catherine Tallon-Baudry

**Affiliations:** ^1^Institut National de la Santé et de la Recherche Médicale-ENS UMR 960Paris, France; ^2^Centre de Recherche de l'Institut du Cerveau et de la Moelle épinière, Université Pierre et Marie Curie-Paris 6, UMR-S975Paris, France; ^3^Centre de Recherche de l'Institut du Cerveau et de la Moelle épinière, INSERM U975Paris, France; ^4^Centre de Recherche de l'Institut du Cerveau et de la Moelle épinière, CNRS UMR 7225Paris, France; ^5^CENIR, Centre de Recherche de l'Institut du Cerveau et de la Moelle épinière, Université Pierre et Marie Curie-Paris 6 UMR-S975, INSERM U975, CNRS UMR 7225Paris, France

**Keywords:** awareness, feature binding, feature selection, LO, MEG, parietal, vision

## Abstract

There is converging evidence that electrophysiological responses over posterior cortical regions in the 200–300 ms range distinguish between physically identical stimuli that reach consciousness or remain unseen. Here, we attempt at determining the sources of this awareness-related activity using magneto-encephalographic (MEG). Fourteen subjects were presented with faint colored gratings at threshold for contrast and reported on each trial whether the grating was seen or unseen. Subjects were primed with a color cue that could be congruent or incongruent with the color of the grating, to probe to what extent two co-localized features (color and orientation) would be bound in consciousness. The contrast between neural responses to seen and unseen physically identical gratings revealed a sustained posterior difference between 190 and 350 ms, thereby replicating prior studies. We further show that the main sources of the awareness-related activity were localized bilaterally on the lateral convexity of the occipito-temporal region, in the Lateral Occipital (LO) complex, as well as in the right posterior infero-temporal region. No activity differentiating seen and unseen trials could be observed in frontal or parietal regions in this latency range, even at lower threshold. Color congruency did not improve grating's detection, and the awareness-related activity was independent from color congruency. However, at the neural level, color congruency was processed differently in grating-present and grating-absent trials. The pattern of results suggests the existence of a neural process of color congruency engaging left parietal regions that is affected by the mere presence of another feature, whether this feature reaches consciousness or not. Altogether, our results reveal an occipital source of visual awareness insensitive to color congruency, and a simultaneous parietal source not engaged in visual awareness, but sensitive to the manipulation of co-localized features.

## Introduction

To characterize neural events leading to visual awareness, many studies adopted the strategy originally proposed by Crick and Koch (Crick and Koch, 1990) and compared neural responses to stimuli that can or cannot be reported. In electrophysiological studies, it is crucial to compare stimuli that are physically strictly identical to avoid any confound due to low-level stimulus properties, such as contrast or duration. There is now converging evidence from various EEG and magneto-encephalographic (MEG) experiments manipulating contrast or masking (for recent reviews, Koivisto and Revonsuo, 2010; Dehaene and Changeux, 2011) that the differential processing of seen and unseen stimuli is notably characterized by an activity over posterior regions, often observed around 200 ms (Koivisto et al., 2005, 2006; Melloni et al., 2011), sometimes later in the case of weakly contrasted stimuli (Wilenius and Revonsuo, 2007), or earlier under strict time constraints (Wyart et al., 2012). This activity appears to be independent from spatial attention and to be directly related to subjective experience (Koivisto et al., 2006; Melloni et al., 2011; Wyart et al., 2012). It can be followed by a more central and more largely distributed activity in the P3 range (Sergent et al., 2005; Del Cul et al., 2007; Wilenius and Revonsuo, 2007).

The posterior activity distinguishing between seen and unseen physically identical stimuli has now been observed by different groups in various experimental conditions and therefore represents a robust electrophysiological correlate of visual awareness. There have been surprisingly few attempts at mapping and localizing the sources of this activity. Vanni et al. (Vanni et al., 1996) found in MEG data that only activity in right Lateral Occipital (LO) regions correlated with subjects' performance in a detection task on masked stimuli, but there was no direct comparison between neural responses to physically identical seen and unseen stimuli. Similarly, Koivisto, and Revonsuo reported posterior sources of awareness-related effects in EEG data but contrasted responses to stimuli that were physically different (Koivisto and Revonsuo, 2010). Sergent et al. (Sergent et al., 2005) reported source localization of EEG responses to physically identical seen and unseen stimuli but focused their analysis on fronto-parietal regions.

**Figure 1:**
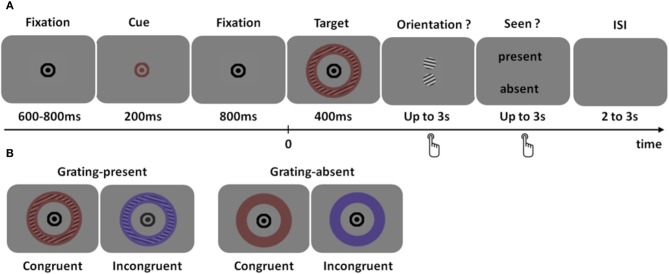
**Experimental design. (A)** Trial time course. After a fixation interval of 600–800 ms, the fixation point briefly changed color, indicating the most likely color of the upcoming stimulus. The target was a colored annulus that contained, in most trials, a faint grating at threshold for consciousness. The color of the stimulus could be identical to that of the cue (congruent trials) or not (incongruent trial). Subjects had to answer two successive questions: first, what was the orientation of the grating (two-alternative forced choice), and second, did the subject detect a grating or not. **(B)** Type of trials. In 87% of the trials, a grating was present in the annulus (left), while it was absent in the remaining 13% grating-absent trials. The color of the target could be identical to that of the cue (congruent trials, 65%) or not (incongruent trials, 35%) Note that the grating modulation has been strongly enhanced for visualization purpose.

We sought to determine more precisely the regions distinguishing between seen and unseen physically identical stimuli using MEG. As depicted in Figure 1, subjects had to detect a faint colored grating, report its orientation in a two-alternative forced choice task (objective task) and decide whether they thought a grating was present or not (subjective task). The same physical stimulus could therefore be classified as seen or unseen, according to subjective reports. Subjective reports were validated by checking that orientation discrimination performance was higher when subjects reported seeing the grating than when subjects reported not seeing it.

The colored grating was preceded by a color cue predictive of the grating color. The color of the stimulus could therefore additionally be classified as congruent or incongruent with the cue. This manipulation was added to probe the extent to which two co-localized features (color and orientation) would be bound in consciousness. Indeed, color and orientation are known to be bound, at least coded in combination together, at an early processing stage and in an unconscious manner (Humphrey and Goodale, 1998; Holcombe and Cavanagh, 2001). Here, we tested whether color congruency would affect the detection of the colored grating.

## Materials and methods

### Subjects

Sixteen volunteers with normal or corrected-to-normal vision took part in the experiment (13 right handed; 10 women; mean age 25 ± 4 years, range 20–30 years old). All had normal color vision as assessed by the Ishihara Color Vision Test. Participants gave written informed consent and were paid for their participation. All procedures were approved by the local ethics committee (Comité pour la Protection des Personnes-CPP, Hôpital de la Pitié-Salpêtrière, Paris, France). Two participants were excluded from the study, one because his head did not fit into the MEG helmet and the other because she failed to comply with the instructions. The data presented here have therefore been obtained in 14 participants.

### Stimuli

Stimulus presentation was controlled by the Psychtoolbox package for Matlab (Brainard, 1997). Stimuli were projected via a mirror system at the center of a screen positioned at 85 cm from subjects' eyes, using a calibrated Mitsubishi X120 projector (resolution, 1024 × 768 pixels; refresh rate, 60 Hz) located outside the shielded recording room. The luminance of the stimuli on the screen was controlled using a Konica Minolta LS-100 luminance meter.

Each target stimulus consisted of a colored annulus, either purple or orange, with inner and outer radii of 2 and 3° of visual angle (hereafter dva), respectively, presented on a gray background (luminance, 15 cd/m2). In grating-present trials, the colored annulus included a grating (spatial frequency five cycles per dva; fall-off from the outer and inner edges of the annulus 0.04 dva). The grating was defined by a faint luminance modulation of the color, giving rise to alternating lighter and darker stripes of the same color. The orientation of the grating was chosen among 20 orientations, equally spaced between 0° and 180°, vertical and horizontal orientations being excluded. The fixation symbol at the center of the screen was composed of a central black disk (0.2 dva) surrounded by a black circle (0.45 dva).

### Task and procedure

Subjects had to answer two questions at each trial: (1) “Which orientation was presented in the target stimulus?” (2) “Do you think a grating was present in the target stimulus or not?”

A trial began with a fixation screen lasting 600–800 ms (Figure 1). The central fixation symbol then turned either orange or purple for 200 ms, indicating the subject the most likely color of the following target. An annulus of the same color as the cue (65% of the trials), or of a different color appeared for 400 ms. In 87% of the trials, the annulus contained a faint grating (grating-present trials) that was set at detection threshold in each subject (see below). In the remaining 13% of the trials, the annulus did not contain any grating (grating-absent trials). Participants were then presented successively with two response screens, which were turned off at response delivery or after 3 s without response. The first response screen displayed two high-contrast grayscale gratings with orientations differing by 60°. In grating-present trials, one of the orientations matched the orientation of the target grating; in grating-absent trials two orientations separated by 60° were randomly chosen. The second response screen displayed the words “present” or “absent”. In both tasks, the two choices were always arranged vertically, above, and below fixation. Subjects answered by pressing the upper or lower response button, respectively with their right index or right middle finger. The upper or lower position of the choices in the response screens was randomly varied from trial to trial to avoid systematic stimulus-response mapping and prevent motor preparation during target presentation. The location of the response options did not overlap the annulus to prevent any masking effect. Participants were explicitly instructed to make a choice even if they were not sure of their responses. During the inter stimulus interval (2–3 s), only a gray background was displayed.

To minimize perceptual differences between the two colors and avoid strong luminance variations at the target onset, we first determined perceptual equiluminance of each color with the gray background in each subject using the heterochromatic flicker method. A colored annulus flickered at 30 Hz against the gray background, and participants were instructed to minimize their sensation of flickering by clicking on two buttons that respectively increased or decreased the luminance of the colored annulus (Wagner and Boynton, 1972). Subjects underwent a total of eight trials (orange or purple, high or low starting luminance, each condition repeated twice) in random order. For each color, we recorded the average luminance at minimal flicker and used these values as luminance parameters in the following sessions. The session lasted about five minutes.

Once the two colors were set at perceptual equiluminance with the background, we determined the contrast at which subjects could detect 50% of grating occurrences, using a “one-up—one-down” staircase procedure. Trials were identical to those described above, except that the contrast of the grating was varied from trial to trial depending on whether the grating was reported as seen or unseen in the previous trial of the same type. Two independent staircases (one for each color) were run, and only the trials of the congruent condition (the most frequent ones) contributed to the staircase convergence. The thresholds thus obtained were used in the following recording sessions.

The staircase session was followed by six recording sessions of the main experimental paradigm described above (mean duration: 8 min/session), during which continuous MEG signals were collected. Each recording session consisted of 92 trials (80 grating-present trials, among which 52 were congruent trials and 28 incongruent trials; and 12 grating-absent trials, among which eight were congruent trials and four incongruent trials).

At the end of the recording session, subjects performed a short (5 min) saccade task in which they made saccade toward targets appearing at 2.5 or 5 dva, along the horizontal, vertical or diagonal directions. This eye-movement calibration session was used to estimate the voltage corresponding to a saccade of 2.5 dva.

### MEG data acquisition

Continuous MEG signals were collected at a sampling rate of 1250 Hz, 0–200 Hz bandwidth, using a whole-head MEG system with 151 axial gradiometers (CTF Systems, Port Coquitlam, BC, Canada) placed in a magnetically shielded room. Head position was monitored before each experimental session using three coils fixed on the subject's head. Vertical and horizontal electro-oculogram (EOG) signals were also collected for offline eye movement rejection.

### MEG data analysis

Data were preprocessed using in-house softwares (http://cogimage.dsi.cnrs.fr/logiciels/). Trials with eye blinks or eye movements exceeding 2.5° of visual angle between 500 ms before cue onset and 500 ms after target onset were excluded from further analysis. Trials with obvious movement or muscle artifacts were also discarded. The remaining grating-present trials were averaged for each subject and each of the following four conditions: seen-congruent, seen-incongruent, unseen-congruent, unseen-incongruent, depending on subjects' report (seen/unseen) and color congruency between cue and target. The respective average number of available trials per subject was 119 (range 35–168, SD = 39.5), 62 (range 25–92, SD = 19.9), 121 (range 84–161, SD = 17.8), and 64 (range 44–95, SD = 13.0). Averaged data were low-pass filtered at 30 Hz, and baseline corrected using the 400 ms preceding cue onset (−1400 to −1000 ms).

To determine the neural correlates of visual awareness, of color congruency and of their possible interaction without any a priori hypothesis, while controlling for multiple comparisons (sensors, time samples), we based our analysis on the cluster-based permutation test proposed by Maris and Oostenveld (Maris and Oostenveld, 2007) which is implemented in the Fieldtrip Toolbox (Oostenveld et al., 2011). We extended this method to repeated-measure ANOVAs. Each sample (one sensor, one time point) of the evoked response to grating-present trials was submitted to a repeated-measure ANOVA with factors Awareness (seen/unseen) and Color Congruency (congruent/incongruent). For each main effect or interaction, samples whose *F*-value exceeded a threshold (here, *F*-values corresponding to a one-tail *p*-value of 0.05) were clustered based on time and space adjacency. Two sensors were considered adjacent if they were separated by less than 4 cm. Each cluster defined in space and time by this procedure was then assigned a cluster-based statistics equal to the sum of the *F*-values of all the samples belonging to the cluster. To test whether this cluster-level statistics could be obtained by chance, the condition labels of the original event-related field (ERF) data of each subject were randomly shuffled. The clustering procedure was then applied on those randomized data, and the maximal cluster *F*-value was measured for each factor and interaction. By repeating the random assignment of condition labels to MEG data 1000 times, we could estimate the distribution of the maximum cluster level *F*-statistics under the null hypothesis, separately for each main effect and each possible interaction. If the original statistic was greater than 95% of the statistic values obtained on randomized data, then the null hypothesis could be rejected with a Monte-Carlo *p*-value < 0.05. Because this method uses the maximum statistics, it intrinsically controls for multiple comparisons.

### Activation strength

The orientation of the magnetic field (outgoing or ingoing) is plotted, by convention, as positive or negative. It does not refer to activation or inhibition, but reflects the orientation of the vector resulting from the sum of local neural current flows with respect to the head surface. To evaluate activation strength independently from the orientation of the flow, we defined an index of activation strength that can be used both at the sensor and source level. Let us consider a region of interest, defined independently by a cluster analysis or a direct contrast between conditions. This region contains sensors (resp. sources) displaying either positive or negative values at the group level. We measured, for each subject and condition, activity across the group-level positive and negative sensors (resp. sources) separately, multiplied by −1 the values obtained over sensor and time points belonging to the negative cluster (resp. sources) and averaged the two values together, to obtain, for each subject and condition, a measure of activation strength that is polarity neutral. For instance, let us consider a cluster of interest, as in Figure 3B. This cluster contains sensors that display a negative difference at the group level (in blue) and sensors that display a positive difference at the group level (in red). To estimate activation strength, we computed, for each subject, two means, one, mean_pos, across sensors, and time points of the positive cluster as defined at the group level, the other, mean_neg, across sensors, and time points of the negative cluster as defined at the group level. Activation strength for each subject was defined as mean_pos + [mean_neg × (−1)]. Note that the activation strength can be either positive or negative depending on the sign of the contrast with respect to the sign of the signal in the four conditions. This measure is similar to the efflux minus influx measure proposed by Hopf and colleagues (Hopf et al., 2006).

### Source localization

Cortical current density mapping was obtained using a distributed model consisting of 15.000 current dipoles. Dipole locations and orientations were loosely constrained to the cortical mantle of a generic brain model built from the standard brain of the Montreal Neurological Institute using the BrainVISA software (http://brainvisa.info). Source localization and surface visualization was performed with BrainStorm (Tadel et al., 2011), which is documented and freely available for download online under the GNU general public license (http://neuroimage.usc.edu/brainstorm). Cortical current maps were computed from the MEG time series using a linear inverse estimator (weighted minimum-norm current estimate), separately for each condition (seen-congruent, seen-incongruent, unseen-congruent, unseen-incongruent,) and for each subject. Cortical currents were then averaged across subjects and over a time window of interest. Awareness-related sources were assessed by computing the *t*-statistic between seen (averaged from seen-congruent and seen-incongruent conditions) and unseen condition (averaged from unseen-congruent and unseen-incongruent conditions). Congruency-related sources were obtained following the same logic. Active sources were defined as those containing at least 15 adjacent vertices whose *t*-value exceeded 3.01, corresponding to a *p*-value of 0.005 (uncorrected for multiple comparisons).

## Results

### Behavior

Subjects reported the presence of a grating in about half of the grating-present trials (detection rate, 50% ± sem 2%), with a low false alarm rate on grating-absent trials (false alarm rate, 10% ± sem 3%), which corresponded to an overall d' of 1.52 ± sem 0.2. The validity of subjective reports was confirmed by objective accuracy measures at the orientation discrimination task: subjects identified the orientation of the grating much more reliably when they reported seeing the grating (orientation discrimination accuracy in seen trials: 91% ± sem 3%; in unseen trials: 52% ± sem 2%, paired *t*-test *p* < 10^−8^). We therefore focused our analysis on those trials in which subjects subjectively saw the grating and discriminated correctly its orientation (hereafter seen trials) and those trials in which subjects did not report seeing the grating (hereafter unseen trials, including both correct and incorrect responses at the orientation discrimination task).

We tested whether color congruency influenced subjects' reports on the presence or absence of the grating, or performance at the orientation discrimination task. None of these measures was affected: d' were similar in congruent and incongruent trials (congruent 1.52 ± 0.20; incongruent 1.41 ± 0.18, paired *t*-test *p* > 0.4). Accuracy at the objective orientation task was not statistically different in congruent and incongruent seen trials (accuracy: congruent 92% ± sem 3%, incongruent 91% ± sem 3%, paired *t*-test *p* > 0.5). Reaction times did not reveal any significant difference either (reaction times of correct responses longer than 0.2 and shorter than 2 s, falling within ± 2 SD: congruent 997 ms ± sem 50 ms, incongruent 1007 ms ± sem 54 ms, paired *t*-test *p* > 0.3). No significant difference between congruent and incongruent unseen trials was observed either (accuracy: congruent 51% ± sem 2%, incongruent 53% ± sem 2%, paired *t*-test *p* > 0.3; reaction time: congruent 763 ms ± sem 60 ms, incongruent 760 ms ± sem 62 ms, paired *t*-test *p* > 0.7).

### Neural correlates of awareness and color congruency

**Figure 2:**
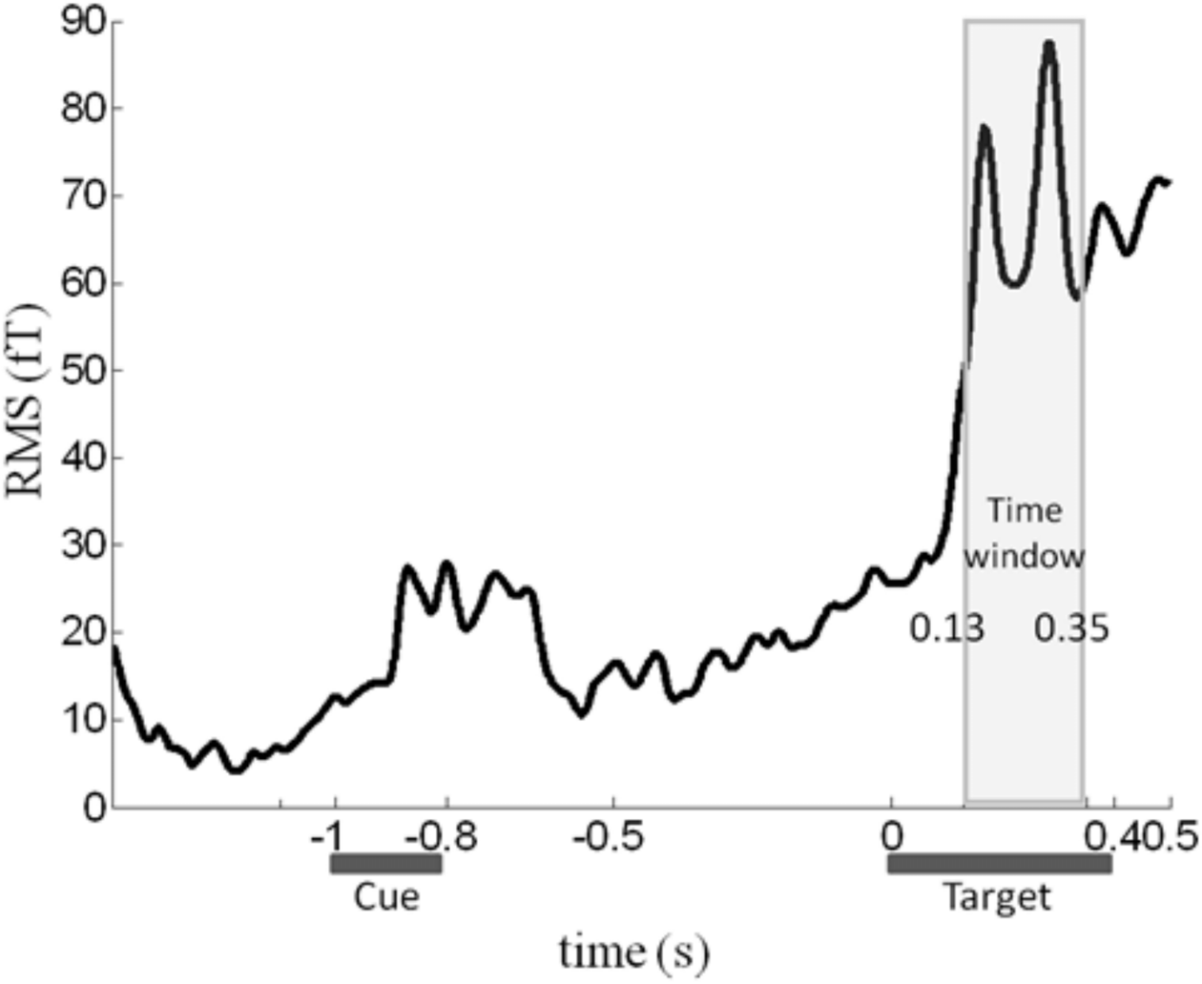
**Time course of the MEG signal.** Root-mean-square (RMS) of the evoked response averaged across all sensors, experimental conditions and subjects. We focused our analysis on the 130–350 ms time window (light gray rectangle), in which a robust response to target onset can be observed.

**Figure 3:**
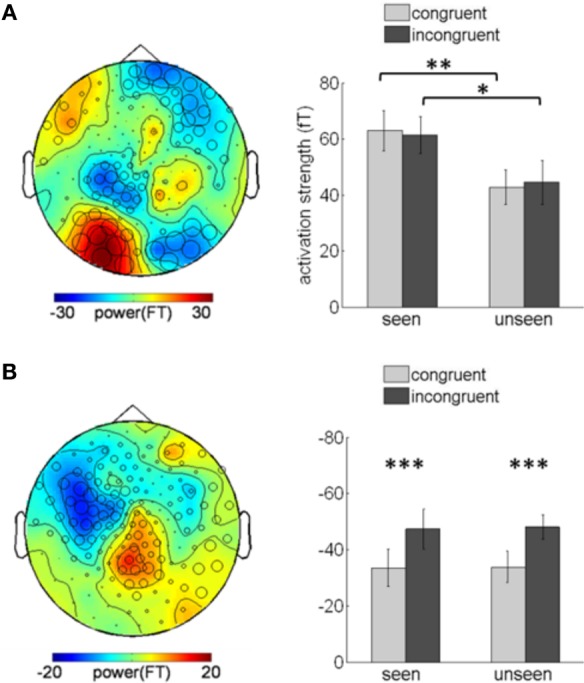
**Awareness and color-congruency related clusters. (A)** Awareness-related effects. Left, topographic map of the difference between seen and unseen gratings, averaged over the 130–350 ms time window. Sensors recruited in the significant awareness-related cluster are indicated by an open circle whose diameter is proportional to the time during which the sensor is involved in the cluster. The maximal difference is observed over occipital sensors. Right, bar graph of activation strength in the awareness-related cluster, plotted separately in the four conditions of interest. A significant awareness effect is observed both in congruent (paired *t*-test, ^**^p < 0.01) and incongruent (^*^p < 0.02) trials. **(B)** Congruency-related effect. Left, topographic map of the difference between congruent and incongruent trials, averaged over the 130–350 ms time-window and distribution of the sensors involved in the congruency-related cluster. The maximal difference is located over left parietal and central sensors. Right, bar graph of the cluster activation strength, plotted separately in the four experimental conditions. A significant difference between congruent and incongruent trials can be observed no matter whether the subject reported seeing the grating or not (paired *t*-tests, congruent vs. incongruent, ^***^p < 0.001). Error bars indicate standard error of the mean.

Although color congruency had no effect on behavior, it might nevertheless have impacted the nature of the neural processes leading to awareness. We therefore analyzed not only the neural correlates of awareness, but also the neural correlates of color congruency and their possible interaction. To perform this analysis without any a priori hypothesis, we used the cluster-based permutation analysis originally proposed by Maris and Oostenveld for *t*-tests (Maris and Oostenveld, 2007) and extended it to ANOVA analyses. This procedure identifies spatio-temporal clusters (neighboring sensors and time points) whose activity significantly varies with a factor or an interaction between factors. It corrects for multiple comparisons in time and space, and does not require defining any a priori spatial or temporal region of interest (see “Materials and Methods” for details). The cluster-based permutation analysis was performed within a time interval from 130 to 350 ms after target onset, which corresponds to the first two peaks of the stimulus-evoked activity (Figure 2). The cluster-based permutation analysis revealed the presence of two significant clusters: one for the main effect of visual awareness (Monte Carlo *P* < 0.05), that presented maximal activity over posterior sensors (Figure 3A, left) and one for the main effect of color congruency (Monte Carlo *P* < 0.05) mainly localized over left parietal and central sensors (Figure 3B, left). No significant cluster was found for the interaction between the factors awareness and color congruency. The smallest *p*-value obtained amongst interaction-related clusters was 0.89.

The significant awareness-related and congruency-related clusters were obtained on ERFs averaged over unequal numbers of trials (e.g., more congruent than incongruent trials). We checked whether an unequal numbers of trials could account for our results by running the following randomization procedure 100 times: (1) for each subject, we pooled all trials of all conditions and created 4 ERFs by randomly partitioning trials in four subsets with the same number of trials as the original four conditions; we thus obtained 4 ERFs with unequal numbers of trials in each condition (2) we computed the summed *F*-statistics on those 4 ERFs, randomly assigned with awareness and congruency labels. Out of the 100 *F*-statistics thus obtained, none was larger than the *F*-statistic obtained for the original awareness and congruency clusters. It therefore seems unlikely that the awareness and congruency effects observed in the original data can be attributed to an unequal number of trials in each condition.

Planned comparisons further confirmed the robustness of the main effect of awareness by showing significant awareness effects in two different sets of trials (congruent and incongruent conditions). We assessed the activation strength of the awareness-related cluster in each subject and condition (Figure 3A, right), separately for congruent and incongruent trials. The difference between seen and unseen trials was significant for both congruent trials (paired *t*-test, *p* < 0.01) and incongruent trials (paired *t*-test, *p* < 0.02). Conversely, activity in the color-congruency cluster was larger for incongruent trials (Figure 3B, right), both for seen and unseen stimuli (paired *t*-test, seen *p* < 0.001; unseen *p* < 0.001), thereby confirming the existence of distinct neural correlates of visual awareness and color congruency.

### Congruency effect in grating-absent trials

**Figure 4:**
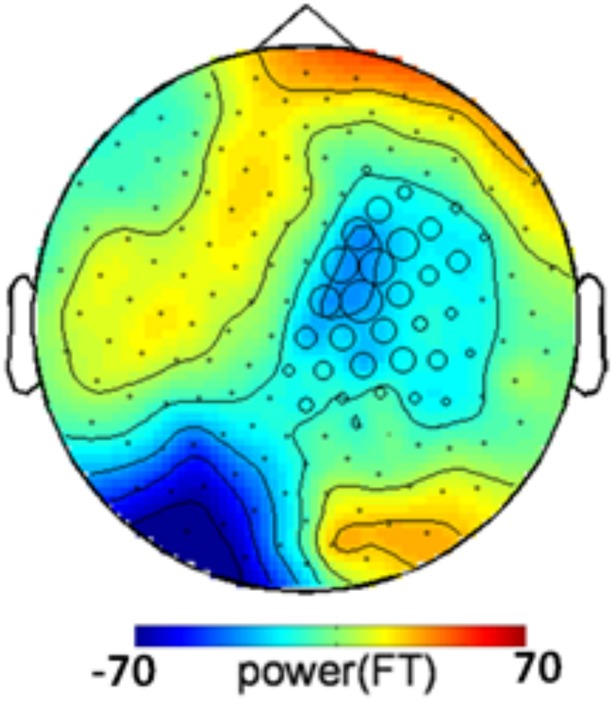
**Congruency-related cluster in grating-absent trials.** Topographic map of the difference between congruent and incongruent grating-absent trials, averaged over the 130–350 ms time window. Sensors recruited in the significant congruency-related cluster in grating-absent trials are indicated by an open circle whose diameter is proportional to the time during which the sensor is involved in the cluster. The topography of the congruency effect in grating-absent trials is different from the one obtained in grating-present trials (Figure 3B).

One could argue that the congruency effect is caused by the color of the annulus supporting the grating rather than by the grating itself. If this were the case, the congruency-related effect should not depend on the presence of a grating in the target, and similar color-congruency effect would be expected in grating-present and grating-absent trials. We therefore analyzed grating-absent trials. Each grating-absent trial was classified as congruent or incongruent based on the relationship between cue color and target color. We ran a cluster-based permutation *t*-test directly derived from (Maris and Oostenveld, 2007) on all grating-absent trials. The threshold used for first-level statistics corresponded to a two-tailed *p*-value of 0.05 and the threshold retained for final statistics, after randomization, was Monte-Carlo *p* < 0.025 (two tailed) to take into account both positive and negative clusters. The procedure revealed a significant congruency-related cluster (Monte-Carlo *p* = 0.02), that was located over right central and parietal sensors (Figure 4). The topography of the congruency-related cluster in grating-absent trials was very different from the topography of the congruency-related cluster in grating-present trials. It therefore seems that color congruency processing relies on distinct neural mechanisms depending on whether the grating is present or not.

### Time course of awareness and congruency effects

**Figure 5:**
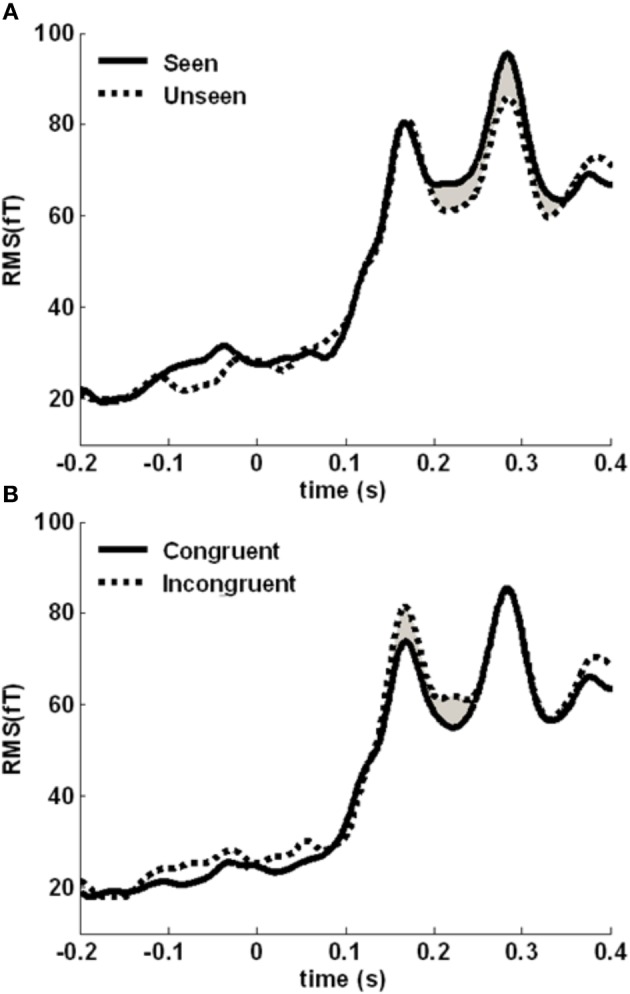
**Time course of the awareness and congruency effects. (A)** Root-mean square (RMS) of the target-evoked response, averaged across the awareness-related cluster, separately for seen (solid line) and unseen (dotted line) gratings. The difference is maximal between 190 and 350 ms (shaded area). **(B)** Averaged RMS across the congruency-related cluster, separately for congruent (solid line) and incongruent (dotted line) trials. The difference is maximal between 150 and 250 ms (shaded area).

We then assessed the time courses of the two main effects. We computed the root-mean-square (RMS) value of the signal across sensors belonging to the cluster, at each time point (Figure 5). The awareness-related difference was quite sustained, in a 190–350 time-window. The congruency effect was more concentrated around the initial peak of activity, between 150 and 250 ms. We focused on these two time-windows to localize the neural sources distinguishing between seen and unseen gratings and between color-congruent and color-incongruent stimuli.

### Source localization

**Figure 6:**
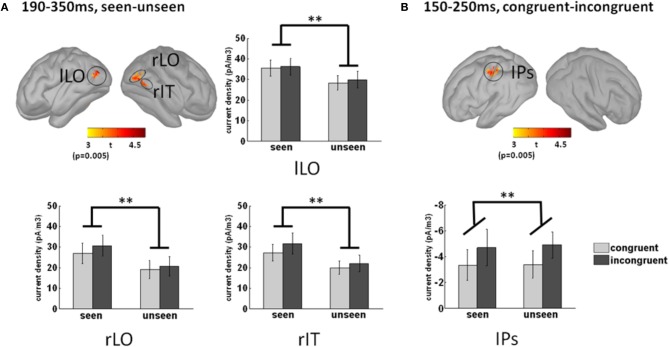
**Source localization of the main effects. (A)** Awareness-related sources were obtained by contrasting seen and unseen trials in the 190–350 ms time-window. Three sources were observed, in the left and right lateral occipital cortices (lLO and rLO respectively), and in the right posterior infero-temporal cortex (rIT). The bar graphs depict activation strength in those three regions in each condition. Activation in those three regions was by definition significantly larger in seen trials than in unseen trials (main effect of awareness, *p* < 0.002). There was neither main effect of congruency (*p* > 0.2) nor interaction (*p* > 0.3) between congruency and awareness. **(B)** Congruency-related sources were obtained by contrasting congruent and incongruent trials in the 150–250 ms time-window. A single significant source was found in the left anterior intraparietal sulcus (aIPS). The bar graph presents aIPS activation strength in each condition. Note that the congruency effect corresponds to greater activation in incongruent trials (main effect of congruency, *p* < 0.01). Neither main effect of awareness (*p* > 0.8) nor interaction between awareness and congruency (*p* > 0.8) could be observed. Error bars indicate standard error of the mean.

To uncover the brain areas contributing to these two main effects, we used a distributed source model. We modeled neural responses to seen and unseen stimuli separately and computed the difference in the 190–350 ms time window, where the main effect was maximal at the sensor level. Significant differences between seen and unseen trials were localized bilaterally in the LO cortex and in the posterior right inferior temporal cortex (Figure 6A). The Talairach coordinates of the three awareness-related activations are presented in Table 1. As depicted in the bar graphs in Figure 6A, those three regions, by definition, showed a significant main effect of awareness (all *p* < 0.002). There was neither interaction with color congruency (all *p* > 0.3) nor main effect of color congruence (all *p* > 0.2).

**Table 1:** **Talairach coordinates (mm) of the activated regions**.

	***x***	***y***	***z***
Left LO (190–350 ms)	−48	−72	−2
Right LO (190–350 ms)	48	−69	−12
Right IT (190–350 ms)	51	−59	−21
Left aIPS (150–250 ms)	−36	41	36

The color-congruency main effect was maximal in the 150–250 ms time window at the sensor level. In this time window, a significant difference was localized in the left intra-parietal sulcus (aIPS), as depicted in Figure 6B. By definition in this region, the main effect of color congruency was significant (*p* < 0.01). Additionally, there was neither interaction with grating awareness (*p* > 0.8) nor main effect of awareness (*p* > 0.8). We further tested whether the physical presence or absence of the grating had an impact on color congruency in left aIPS. For each subject, we computed activation strength in left aIPS in congruent/incongruent and grating-present/grating-absent trials. To equate subjective reports, we restricted this analysis to trials in which subjects reported no stimulus, i.e., unseen grating-present trials and correctly-rejected grating-absent trials. A Two-Way repeated measure Anova with the factors color congruency and the physical presence or absence of a grating revealed a significant interaction between the two factors (*p* < 0.04; main effects, all *p* > 0.25). This interaction corresponded to a significant congruency effect in unseen grating-present trials and no significant congruency effect in correctly-rejected grating-absent trials (*post-hoc* paired *t*-test, respectively *p* < 0.02 and *p* > 0.15). In other words, the color-congruency effect in aIPS was dependent on the physical presence of the grating.

In the analysis presented above, a region was considered differentially active if it contained at least 15 adjacent vertices (out of 15.000) with an individual *p*-value smaller than 0.005, uncorrected for multiple comparisons. With a more liberal threshold (10 adjacent vertices with *p* < 0.01), the sources of the awareness-related effect spread both posteriorly and anteriorly in the occipital and temporal lobes, especially in the right hemisphere. An additional awareness-related focus appeared in the right superior temporal sulcus. No activation was found in frontal or parietal regions. Lowering the threshold in the congruent vs. incongruent contrast revealed an additional small focus of activity in right aIPS. It also revealed small regions of differential activation in right motor regions and in the left inferior frontal sulcus. In other words, the use of a more liberal threshold did not alter much the results. In particular, it did not reveal any awareness-related activation in the fronto-parietal network or congruency-related activations in the ventral visual pathway.

## Discussion

This experiment confirmed that perceiving or not the same visual stimulus at threshold is associated with a sustained posterior activity between 200 and 300 ms. This activity correlated with subjective experience and was not influenced by color congruency, as revealed by using an ANOVA cluster permutation test. We could further localize the sources of this posterior awareness-related activity bilaterally in the left and right LO regions as well as in the right posterior infero-temporal region. Behavioral data did not reveal any interference between color congruency and grating detection. The neural processing of color congruency was independent from grating conscious detection, but depended on the physical presence of the grating.

Our results confirm previous EEG studies linking LO activity in the 200–300 ms latency range with visual awareness, thereby ruling out the possibility that in those studies LO activity was related to physical differences between stimuli (Vanni et al., 1996; Koivisto and Revonsuo, 2010). Gamma-band oscillations discriminating seen from unseen gratings (Wyart and Tallon-Baudry, 2008) were also source-localized in LO (Wyart and Tallon-Baudry, 2009). This good convergence of electrophysiological markers is not trivial, considered that evoked responses and gamma-band oscillations are not necessarily co-localized in those regions of the visual system (Tallon-Baudry et al., 2005) and that two very different methods of source localization were used in the two studies (minimum-norm estimate on evoked responses in the present report, beamformer on gamma-band oscillations). This localization is in good agreement with fMRI studies that underlined the critical role of LO in explicit objective recognition of familiar objects (Grill-Spector et al., 2000) or letters (Kleinschmidt et al., 2002), and more recently in subjective visual experience (Hesselmann et al., 2011).

We did not observe any awareness-related frontal or parietal activation. Obviously absence of proof is not proof of absence. In particular, activity in frontal regions may have been more difficult to detect than activity in posterior regions: since subjects were seated with the back of their head resting on the MEG helmet, frontal regions were farther away from the sensors than posterior ones. However, this sensitivity argument does not hold for parietal regions since we could successfully identify sources in aIPS. This parietal region was not related with awareness but with color congruency, in line with previous studies on color priming (Kristjansson et al., 2007) and on attention to color (Liu et al., 2011). Parietal activations have been related to visual awareness in fMRI studies using rivalry (Kleinschmidt et al., 1998; Lumer et al., 1998), change detection (Beck et al., 2001), or continuous flash suppression (Hesselmann et al., 2011) paradigms. However, those results have been questioned: left parietal regions do not seem to play a causal role in visual awareness, as revealed by a TMS study (Beck et al., 2006), and parietal activity could be related to perceptual processes rather than to awareness *per se* (Kanai et al., 2010). In line with those studies, aIPS activation appeared to be independent from awareness in the present experiment.

Color congruency did not affect the perception of the grating, and the color-congruency effect displayed in aIPS was independent of grating awareness. This pattern of results could indicate that color and orientation were treated independently, even if the two features were co-localized. However, our results indicate a more subtle pattern of interaction between color and orientation. Indeed, the topography of the color congruency effect was different in grating-present and grating-absent trials, and no color congruency effect was observed in aIPS for grating-absent trials. These results suggest that at some point, color, and orientation were bound, since the presence of an oriented grating affected color congruency processing. This interpretation is in line with previous results showing that co-localized orientation and color information are processed jointly at an early processing stage (Humphrey and Goodale, 1998; Holcombe and Cavanagh, 2001). Left aIPS activity could therefore represent an active de-grouping process of color and orientation information to select color and analyze color congruency, a process that would be present only in grating-present trials and more pronounced when an incongruent, distracting color is presented. Because aIPS modulation was present in both seen and unseen trials, this process does not appear to depend on stimulus awareness. This interpretation, although tentative, is in line with previous findings specifically relating left aIPS activation with responses to weak targets in the presence of salient distractors (Mevorach et al., 2009). Our results therefore suggest the existence of an early grouping of co-localized features followed by a feature selection process, independent of feature awareness, involving left parietal regions.

### Conflict of interest statement

The authors declare that the research was conducted in the absence of any commercial or financial relationships that could be construed as a potential conflict of interest.
